# Study of the Ability of Bifidobacteria of Human Origin to Prevent and Treat Rotavirus Infection Using Colonic Cell and Mouse Models

**DOI:** 10.1371/journal.pone.0164512

**Published:** 2016-10-11

**Authors:** Mélanie Gagnon, Allison Vimont, André Darveau, Ismaïl Fliss, Julie Jean

**Affiliations:** 1 Institute of Nutrition and Functional Foods, Department of Food Science, Laval University, Quebec, Quebec, Canada; 2 Department of Biochemistry, Microbiology and Bioinformatics, Laval University, Quebec, Quebec, Canada; University of Liverpool, UNITED KINGDOM

## Abstract

Rotavirus is the leading cause of severe acute gastroenteritis among children worldwide. Despite effective vaccines, inexpensive alternatives such as probiotics are needed. The aim of this study was to assess the ability of probiotic candidate *Bifidobacterium thermophilum* RBL67 to inhibit rotavirus infection. Bacterial adhesion to intestinal cells and interference with viral attachment were evaluated *in vitro*. *B*. *thermophilum* RBL67 displayed adhesion indexes of 625 ± 84 and 1958 ± 318 on Caco-2 and HT-29 cells respectively and was comparable or superior to four other bifidobacteria, including *B*. *longum* ATCC 15707 and *B*. *pseudolongum* ATCC 25526 strains. Incubation of *B*. *thermophilum* RBL67 for 30 min before (exclusion) and simultaneously (competition) with human rotavirus strain Wa decreased virus attachment by 2.0 ± 0.1 and 1.5 ± 0.1 log_10_ (by 99.0% and 96.8% respectively). Displacement of virus already present was negligible. In CD-1 suckling mice fed *B*. *thermophilum* RBL67 challenged with simian rotavirus SA-11, pre-infection feeding with RBL 67 was more effective than post-infection feeding, reducing the duration of diarrhea, limiting epithelial lesions, reducing viral replication in the intestine, accelerating recovery, and stimulating the humoral specific IgG and IgM response, without inducing any adverse effect. *B*. *thermophilum* RBL67 had little effect on intestinal IgA titer. These results suggest that humoral immunoglobulin might provide protection against the virus and that *B*. *thermophilum* RBL67 has potential as a probiotic able to inhibit rotavirus infection and ultimately reduce its spread.

## Introduction

Human rotavirus is the leading cause of severe dehydrating diarrhea in infants and young children worldwide, in both developed and developing countries. Peak incidence occurs in children 2 years of age and under, with an estimated 0.3 rotavirus-induced gastroenteritis episode per child-year [1]. Between 1990 and 2011, rotavirus infection caused an estimated 197,000 deaths per year, or 23 per hour [2]. About 90% of these occurred in low-income countries in Africa and Asia and were associated with poor health care [3]. The virus is transmitted primarily via the fecal-oral route and to a lesser extent via vomit, spreading via contaminated food or water, direct person-to-person contact, aerosols, and environmental surfaces [4]. Infectious doses as low as one plaque-forming unit [5], viral loads as high as 10^12^ particles per gram in feces and vomit [4] and persistence on fomites and hands [6, 7] all contribute to the high incidence of rotavirus illness. Since the spread of the virus is very difficult to control, rotavirus outbreaks occur often in crowded locations such as daycare centers, hospitals and schools [4]. Rotavirus infects primarily mature enterocytes in the intestinal epithelium, leading to malabsorption and osmotic diarrhea [8, 9].

Since no specific anti-rotavirus therapy is currently available, the main treatment is fluid replacement to prevent dehydration and zinc treatment to decrease the severity and duration of the diarrhea [3]. Two effective rotavirus vaccines, namely RotaTeq^®^ (Merck and Co) and Rotarix^®^ (GSK Biologicals), have been available since 2006 and are recommended by the World Health Organization for use in all countries, particularly in those where diarrhea-related mortality in children younger than 5 years is common [3]. The number of countries that have introduced rotavirus vaccines increased from 5 in 2011 to 35 in 2015 [10]. Both vaccines have been reported to be highly effective in high-income settings [11]. Nevertheless, the protection afforded by these live oral vaccines is reduced in low-income settings [12]. Consequently, inexpensive and effective supplementary or complementary therapies remain necessary.

The role of intestinal microbiota in modulating enteric viral infections has been highlighted by several recent studies [13, 14], in particular with norovirus [15] and rotavirus [16]. In this context, the use of probiotic strains as an alternative therapy has been proposed [17, 18]. Based on consultation with scientific experts, the World Health Organization in 2001 retained the following (and current) definition of probiotics: “live microorganisms that, when administered in adequate amounts, confer a health benefit on the host” [19]. General benefits associated with probiotics include support of a healthy gut microbiota, a healthy digestive tract and a healthy immune system [20]. More precisely, some probiotic strains have been shown to stimulate gut epithelial cell proliferation significantly, to reduce gut permeability and to enhance immune responses as well as providing other health benefits [21–23]. Decreased duration, severity or incidence of infantile diarrhea has been noted in some pediatric clinical trials in conjunction with rotavirus outbreaks. Several probiotic strains have been tested, including the Gram-positive strains of *Bifidobacterium animalis* subsp. *lactis* Bb12 [24], *Lactobacillus rhamnosus* GG [25, 26], *Lb*. *paracasei* [27], *B*. *bifidum*, *Streptococcus thermophilus* [28], and recently the Gram-negative *Escherichia coli* Nissle [29]. The beneficial effects of probiotics are thought to be bacterial species specific, although the precise mechanisms involved in these effects remain largely unknown [27, 30], thus calling for the use of *in vitro* and *in vivo* models.

The probiotic candidate *Bifidobacterium thermophilum* RBL67 used in this study was isolated previously from stool samples obtained from breast-fed infants during research conducted by our group [31, 32]. Such infants are known to be less affected by infections such as gastroenteritis [33]. The safety of bifidobacteria for use as probiotics is presumed on the basis of the long historical consumption of fermented milks and ever-growing knowledge on the taxonomy and physiology of the bacteria they contain [34, 35]. Moderately tolerant of oxygen and low pH [32], *B*. *thermophilum* RBL67 appears resistant to only one antibiotic (nalidixic acid), and is sensitive to nine antibiotics including ampicillin, tetracycline and chloramphenicol [36]. It reportedly produces a bacteriocin-like substance active against *Listeria* and *Salmonella* [31, 32, 37, 38] and protects epithelial integrity upon *Salmonella* challenge in the presence of competing human microbiota [39]. Its strong anti-*Salmonella* activity has been demonstrated in an *in vitro* continuous fermentation system simulating the juvenile intestinal ecosystem, along with its ability to rebalance the metabolic activity of gut microbiota after antibiotic treatment [38]. Its genome has been sequenced [40] and is available for genetic studies.

The aim of the present study was to evaluate the adhesiveness of *B*. *thermophilum* RBL67 to Caco-2 and HT-29 cells as well as its ability to interfere with rotavirus attachment. An *in vivo* study using suckling mice was also performed to assess its host-protection properties under intestinal conditions and its impact on the course of rotavirus infection, including diarrhea, virus replication, colon histology and the immune response.

## Materials and Methods

### Bacterial strains

Three strains of *Bifidobacterium* from the Research Network on Lactic Acid Bacteria (RBL Network) were used in this study, namely *B*. *thermophilum* RBL67, *B*. *thermacidophilum* RBL69 and *B*. *thermacidophilum* RBL70, isolated from stool samples obtained from infants [31, 32] and preselected on the basis of resistance to gastrointestinal conditions. Two *Bifidobacterium* strains obtained from the American Type Culture Collection were used for comparison purposes: *B*. *longum* ATCC 15707 and *B*. *pseudolongum* ATCC 25526. All strains were cultured in MRS agar supplemented with 0.05% (wt/vol) L-cysteine-hydrochloride at 37°C under anaerobic conditions as described previously [41] and enumerated (in colony-forming units, cfu) on Beerens agar plates [42].

### Cell cultures

All cell lines were cultured in Gibco media (Invitrogen, Burlington, Ontario, Canada) at 37°C under a 5% CO_2_ atmosphere. Caco-2 cells (ATCC HTB-37) were grown routinely in Dulbecco’s Modified Eagle medium as described previously [41]. HT-29 cells (ATCC HTB-38) were grown routinely in RPMI 1640 medium supplemented with 10% fetal bovine serum (FBS), 2 mmol/L L-glutamine, 100 U/mL penicillin and 100 μg/mL streptomycin. Rhesus monkey kidney cell line MA-104 (ATCC CRL-2378.1) was routinely grown in Eagle’s minimal essential medium supplemented with 10% FBS, 2 mmol/L L-glutamine, 1% nonessential amino acids, 1% HEPES buffer, 1.125 g/L sodium bicarbonate, 100 U/mL penicillin and 100 μg/mL streptomycin. For adhesion and inhibition assays, monolayers of human colon carcinoma cells (Caco-2 and HT-29) were seeded at a density of 10^4^ cells per well in 24-well tissue culture plates (Falcon, Becton Dickinson and Co., Franklin Lakes, NJ). The culture medium was replaced every day, and the monolayer was used at post-confluence (10^6^ cells/well) after 15 and 21 days for Caco-2 and HT-29 respectively. The medium was replaced with its antibiotic-free equivalent 18 h before performing the assays.

### Viruses

Human rotavirus strain Wa (ATCC VR-2018) and simian rotavirus strain SA-11 (ATCC VR-1565) used for *in vitro* and *in vivo* assays, respectively, were propagated in MA-104 cells as described previously [6] with some modifications. Briefly, confluent cell monolayers were infected at an MOI of 1.5 with rotavirus pre-activated with 16 μg/mL of porcine trypsin (Sigma Chemical Co., St. Louis, MO) for 30 min at room temperature. After adsorption (1 h at 37°C), maintenance medium (MEM + 2% FBS) was added and cells were incubated for 48 h at 37°C with 5% CO_2_. The infected cultures were then frozen (-80°C) and thawed (37°C) three times, the contents centrifuged at 1,000 x g for 10 min and the supernatant dispensed into vials for storage at -80°C until use. Infectious viruses were quantified using the following cell based methods. Strain Wa titer was determined from a cell culture immunofluorescence assay [43] and expressed in focus-forming units (ffu) per mL. The titer of simian rotavirus strain SA-11 was determined by plaque assay [44] with some modifications and expressed in plaque-forming units (pfu) per mL. Briefly, confluent MA-104 cells in 6-well plates were washed with phosphate-buffered saline (PBS) and infected with 100 μL of pre-activated SA-11 at 37°C for 1 h in a 5% CO_2_ atmosphere. Monolayers were then overlaid with MEM supplemented with 4% FBS, 1.2% agarose type II (Sigma) and 6.5 μg/mL trypsin. Plates were incubated for 3 days at 37°C (5% CO_2_), fixed in 3.7% (v/v) formaldehyde and then stained with 0.1% (w/v) aqueous crystal violet solution to reveal lytic plaques resulting from the cytopathic effect.

### *In vitro* adhesion of *Bifidobacterium* strains to Caco-2 and HT-29 cells

Cells monolayers were washed twice with sterile PBS, drained, contacted with 250 μL of *Bifidobacterium* suspension (5×10^9^ cfu/mL in PBS) per well, incubated for 60 min at 37°C under anaerobic conditions, then rinsed with PBS. Adherent bacteria were then collected in three washings of the monolayer with PBS after 15 min of trypsin-EDTA treatment at 37°C and enumerated by plating. Results are expressed as the adhesion index, which is the number of bacteria adhering per 100 cells.

### *In vitro* antagonism against rotavirus attachment to Caco-2 and HT-29 cells

Cell monolayers were washed twice with sterile PBS, drained, then contacted with 250 μL of pre-activated human rotavirus strain Wa suspension (5×10^6^ ffu/mL) per well for 30 min after (exclusion), simultaneously with (competition) or 30 min before (displacement) the addition of 250 μL of *B*. *thermophilum* RBL67 suspension (5×10^9^ cfu/mL) per well. Positive controls received PBS instead of *B*. *thermophilum* RBL67. Cells were then incubated for 90 min at 37°C under anaerobic conditions, rinsed with PBS, and attached virus was collected in three washings with PBS by repeated pipetting after 15 min of trypsin-EDTA treatment at 37°C. Attached virus was enumerated by cell culture immunofluorescence assay as described previously [43].

### Mice

Timed pregnant CD-1 mice obtained from Charles River (St-Constant, Québec, Canada) were fed a standard laboratory rodent chow (Charles River Laboratories, Wilmington, MA), provided with water ad libitum, and kept in an animal room maintained at 22 ± 2°C with a 12 h light/dark cycle. Each dam and its litter were housed in a micro-isolator cage (Lab Products, Seaford, DE) on a cage ventilation rack (Lab Products) that provided a unidirectional flow of filtered air over the cage hoods. All dams were rotavirus antibody negative as measured by enzyme-linked immunosorbent assay (ELISA, described below). The experiments were performed with the approval of the animal care committee of Laval University “Comités de protection des animaux” (SIRUL number: 76543) and the mice were cared for and handled in conformity with the Canadian Guide for the Care and Use of Laboratory Animals [45]. No animal died during the study except for the euthanasia scheduled for the taking of biological samples.

### Suckling mice treatment groups

Three experimental repetitions were done using pups from the litters of fifteen dams (10–14 pups per litter, total n = 189). Experiments were conducted on suckling mice because preliminary assays shown that adults mice are highly resistant to rotavirus infection and required either antibiotic pretreatment or a genetically modified mice model. Dams and their litters were assigned randomly to one of the five experimental groups (Table 1). *B*. *thermophilum* RBL67 was given orally at a daily dose of 1×10^9^ cfu in 20 μL of PBS for 7 consecutive days, from the age of 3 days or 9 days depending on the experimental group. On day 9, simian rotavirus strain SA-11 not trypsin-activated was given orally at a single dose of 1×10^4^ pfu in 20 μL of PBS with 10% blue food coloring (McCormick Co., London, Ontario, Canada) added as a tracer instead of human rotavirus Wa which is noninfectious to mice. Control animals received 20 μL of placebo (PBS with 10% blue food coloring). After each feeding and infection, the pups were returned to their mothers and allowed to suckle. Activity level, body weight and external signs of disease (diarrhea, dehydration) of mice were observed daily over the course of experiment. Mice were sacrificed under anesthesia by intraperitoneal injection of 0.01 mL/g of a ketamine/xylazine mixture (respectively 15 mg/mL and 1 mg/ml) for microbial and histological analysis and measurement of the immune response. All efforts were made to minimize animal suffering.

**Table 1:** Distribution of experimental groups in the *in vivo* assay.

	*B*. *thermophilum* RBL67		Simian rotavirus strain SA-11
Group	Feeding period (days)	Daily dose (cfu/mouse)	Inoculum on day 9 (pfu/mouse)
A	-	-	Control treatment
B	3–9	10^9^	Control treatment
C	-	-	10^4^
D	3–9	10^9^	10^4^
E	9–15	10^9^	10^4^

### Enumeration of intestinal bifidobacteria and rotavirus

The intestines were removed aseptically from the sacrificed mice, weighed, placed in 1.0 mL of PBS with 5% antibiotic solution (for viral counts) or 0.5 g/L L-cysteine (for bacterial counts) and homogenized with an Ultra-Turrax (Janke and Kunkel, Staufen, Germany) on ice for 15 s at 13,500 rpm. For viral quantification, the suspension was then centrifuged at 13,500 rpm for 5 min to remove debris and frozen at -20°C until plaque assay. For bacterial quantification, the suspension was diluted immediately, plated on Beerens selective agar and incubated at 37°C for 72 h under anaerobic conditions. Isolates were then confirmed by Gram staining to be Gram-positive rods and identified using API 50CH galleries (BioMérieux, Montréal, Québec, Canada) according to the manufacturer’s instructions.

### Histological analysis of the colon

Sections of colon were fixed in PBS containing 4% (w/v) paraformaldehyde (TAAB, Callera Park, Aldermaston Berks, England), embedded in paraffin, cut into 5 μm slices using a microtome (Model 2040, Reichert-Jung, Vienna, Austria) and stained with hematoxylin and eosin. A pathologist kept unaware of sample origin then examined the epithelium morphology using a light microscope (Leica Microsystems, Richmond Hill, Ontario, Canada). Images were acquired by a Hyper HAD camera (Sony Ltd., Willowdale, Ontario, Canada) and Image Matrox Inspector software 3.1 (Matrox Electronic Systems Ltd., Dorval, Québec, Canada).

### Quantification of the immune response in intestinal and serum samples by ELISA

The level of intestinal rotavirus-specific IgA was determined by ELISA as described previously [46, 47] with some modifications. Briefly, simian rotavirus strain SA-11 was fixed in a 96-well plate (8×10^5^ pfu/mL in 2% formaldehyde-PBS solution). Diluted intestinal homogenate (500 mg/mL) was added, followed by peroxidase-labeled goat anti-mouse IgA and finally the O-phenylenediamine (OPD, Sigma) standard solution in the presence of H_2_O_2_. Optical density (OD) was measured at 450 nm on a Thermomax microplate reader (Molecular Devices, Opti-Ressources, Charny, Québec, Canada). Rotavirus-specific IgG and IgM in serum obtained by centrifuging (4,000 × g for 10 min at 4°C) cardiac blood from individual mice were determined using the same protocol except that peroxidase-conjugated goat anti-mouse (H+L, Kirkegaard and Perry Laboratories) was used. Intestinal fluid and serum from non-infected mice not fed *Bifidobacterium* were used as negative controls on each plate. Titers were calculated as log_10_ (1/Dc) where the cut-off dilution (Dc) was the dilution yielding twice the absorbance of the negative controls.

### Statistical analyses

Adhesion indexes of *Bifidobacterium* strains and attachment of human rotavirus strain Wa to intestinal cells were compared among treatments using the one-way analysis of variance (ANOVA) general linear model followed by Tukey’s HSD test. Body weight, simian rotavirus strain SA-11 titer, intestinal *B*. *thermophilum* RBL67 count and immunoglobulin concentration were compared among treatment groups using the one-way ANOVA general linear model followed by the Hsu-Dunnett test. Statistical significance was declared at *P* < 0.05. All statistical analyses were performed using JMP^®^ software version 10.0 (SAS Institute Inc, Cary, NC).

## Results

### *B*. *thermophilum* RBL67 displayed high adhesion ability to intestinal cells

**Fig 1:**
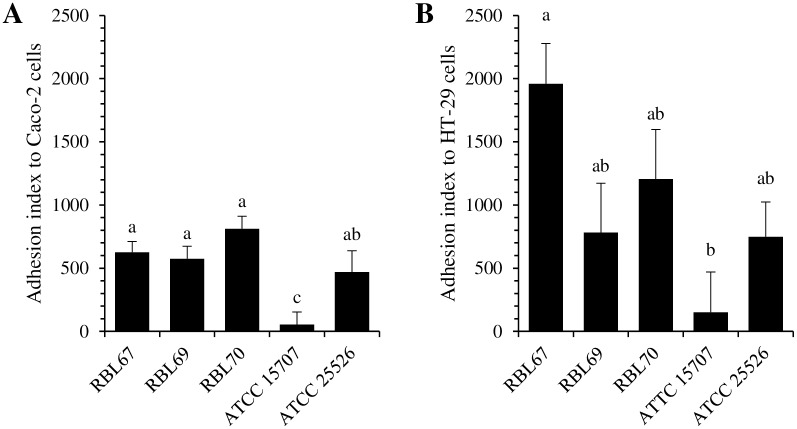
Adhesion of *Bifidobacterium* strains RBL67 (*thermophilum*), RBL69 and RBL70 (*thermacidophilum*), ATCC 15707 (*longum*), and ATCC 25526 (*pseudolongum*) to cultured intestinal cell monolayers: (A) Caco-2, (B) HT-29. Adherent bacteria were counted by plating after 1 h of contact. Index is the number per 100 intestinal cells. Error bars indicate the standard error of the mean. Different letters indicate significant difference between assays (Tukey’s HSD test, *P* < 0.05, n = 3).

The ability of *B*. *thermophilum* RBL67 to adhere to Caco-2 and HT-29 cells was compared with that of four other *Bifidobacterium* strains including two *B*. *thermacidophilum* strains isolated from infant stool samples (RBL69 and RBL70) and two reference strains, namely *B*. *longum* ATCC 15707 and *B*. *pseudolongum* ATCC 25526 (Fig 1), recognized for their weak and strong adhesion respectively [48]. The adhesion index of *B*. *thermophilum* RBL67 was evaluated at 625 ± 84 and 1958 ± 318 on Caco-2 and HT-29 respectively. Expressed per unit area, *B*. *thermophilum* RBL67 adhesion was 3.3 ± 0.4 x 10^4^ cfu/mm² and 1.0 ± 0.2 x 10^5^ cfu/mm² to Caco-2 and HT-29 respectively. No significant difference was noted between *B*. *thermophilum* RBL67, RBL69, RBL70 and *B*. *pseudolongum* ATCC 25526. As expected, *B*. *longum* ATCC 15707 showed significantly lower adhesion indexes of 54 ± 97 and 150 ± 318 respectively on Caco-2 and HT-29.

### *B*. *thermophilum* RBL67 decreased rotavirus attachment to intestinal cells *in vitro*

**Fig 2:**
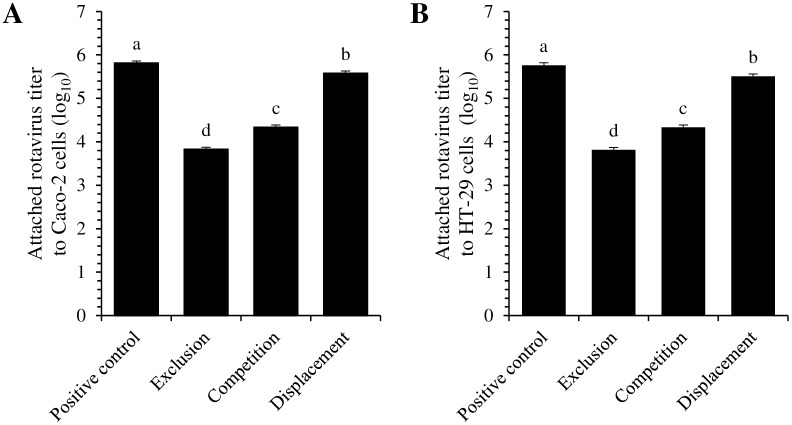
Attachment of human rotavirus strain Wa to Caco-2 (A) and HT-29 (B) cells in exclusion, competition and displacement assays with *B*. *thermophilum* RBL67. Positive controls were contacted with rotavirus only. Attached rotavirus titer was measured by immunofluorescence after 1.5 h of contact and expressed in focus-forming units per mm². Error bars indicate the standard error of the mean. Different letters indicate significant difference between assays (Tukey’s HSD test, *P* < 0.05, n = 3).

Inhibition of rotavirus attachment to intestinal cells by *B*. *thermophilum* RBL67 was evaluated using exclusion, competition and displacement assays (Fig 2). Preliminary 48-h trials indicated that the highest attachment to both intestinal cell lines was obtained after 1.5 h of contact time (S1 Fig). When rotavirus was added alone (positive control), 5.8 ± 0.1 and 5.7 ± 0.1 log_10_ were attached to Caco-2 and HT-29 cell lines respectively. However, when rotavirus was added 30 min after the addition of *B*. *thermophilum* RBL67 (exclusion), the number of viruses attached dropped by two log cycles (to 3.8 ± 0.1). No significant difference between cell lines was observed (*P* > 0.05). In competition and displacement assays, rotavirus attachment was respectively 4.3 ± 0.1 log_10_ and 5.6 ± 0.1 log_10_.

### Intestinal concentration of *Bifidobacterium* strain RBL67 was not influenced by rotavirus infection

**Fig 3:**
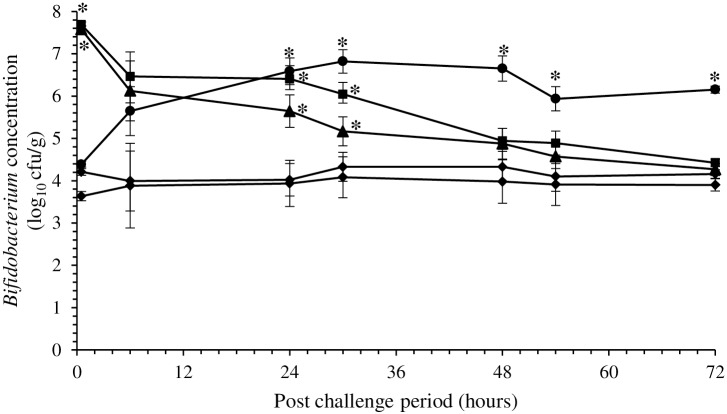
*Bifidobacterium* viable counts over a 72 h period in the intestinal contents of suckling CD-1 mice: not fed strain RBL67 (group A, ◆), fed strain RBL67 (group B, ▴), not fed strain RBL67, challenged with rotavirus SA-11 (group C, ✕), fed strain RBL67 before challenge (group D, ■), fed strain RBL67 after challenge (group E, ●) (See Table 1 for details) *Bifidobacterium* counts were determined by plating. Error bars indicate the standard error of the mean. An asterisk indicates significant difference compared to **◆** (Hsu-Dunnett test, *P* < 0.05, n = 3).

The protective effect of *B*. *thermophilum* RBL67 observed *in vitro* was evaluated in suckling CD-1 mice distributed to five experimental groups (A to E, Table 1). Viable counts of *Bifidobacterium* in the intestinal contents were monitored for 72 h (Fig 3). In control mice (group A), bifidobacteria were detected at about 3.8 log_10_ cfu/g, which remained stable throughout the experiment. Similar results (P > 0.05) were obtained with mice challenged with rotavirus (group C). In mice fed *B*. *thermophilum* RBL67 then challenged with rotavirus (group D), *Bifidobacterium* counts gradually decreased from 7.5 to 4.2 log_10_ cfu/g and were no longer significantly different from control mice after 48 h. In contrast, counts increased from 4.2 to 6.6 log_10_ cfu/g in mice that received *B*. *thermophilum* RBL67 for 7 days after challenge (group E) and were significantly higher than in control mice starting at 24 h.

### Bifidobacterium strain RBL67 fed prior to challenge reduced diarrhea duration

Control mice (group A) did not develop diarrhea at any point. Mice challenged with the simian rotavirus strain SA-11 without receiving *B*. *thermophilum* RBL67 (group C) suffered from diarrhea beginning at 24–30 h post challenge and most intensely at 54 h. Reduced activity was observed during the 7 days post challenge. In contrast, mice that received *B*. *thermophilum* RBL67 for 7 days prior to challenge (group D) suffered from diarrhea later (starting at 48 h) and recovered earlier than did group C mice. The post challenge feeding with *B*. *thermophilum* RBL67 (group E) also delayed the onset of diarrhea slightly (30 h) without accelerating recovery. Body weight was significantly lower in groups C, D and E during the first 72 h post challenge (S2 Fig).

### Pre-challenge feeding with *B*. *thermophilum* RBL67 limited intestinal replication of rotavirus

**Fig 4:**
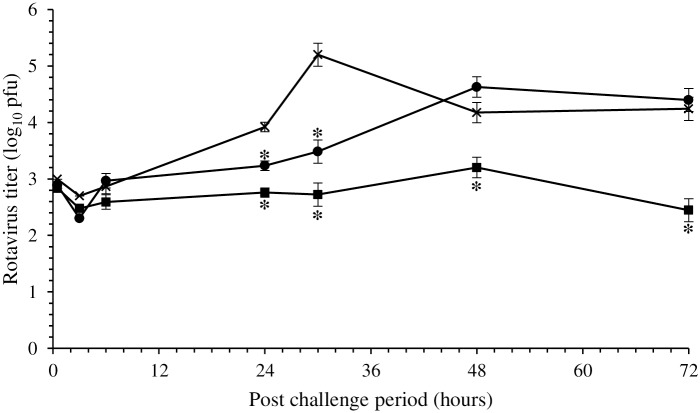
Simian rotavirus strain SA-11 titer measured in the intestinal contents of suckling CD-1 mice: challenged with the virus (group C, ✕), fed *Bifidobacterium* strain RBL67 before challenge (group D, ■), fed strain RBL67 after challenge (group E, ●). Rotavirus titer was determined by plaque assay. Error bars indicate the standard error of the mean. An asterisk indicates statistical significance compared to **✕** (Hsu-Dunnett test, *P* < 0.05, n = 3).

The course of rotavirus infection in suckling CD-1 mice was observed for 72 h by measuring strain SA-11 titer in the intestinal contents using ELISA (Fig 4). No rotavirus was detected in control mice (group A) or mice fed *B*. *thermophilum* RBL67 (group B). In mice not fed *B*. *thermophilum* RBL67 but challenged with strain SA-11 (group C), the titer increased from 2.9 log_10_ initially, peaked to 5.2 log_10_ after 24 h and decreased at 4.2 log_10_ after 30 h, as described previously [49]. In mice that received *B*. *thermophilum* RBL67 for 7 days after challenge (group E), a 30 h delay in the increase from the initial titer (3.5 log_10_) was noted. The titer then rose to 4.4 log_10_ at 72 h. Pre-challenge feeding with *B*. *thermophilum* RBL67 (group D) appeared to control the infection, limiting the rotavirus titer to between 3.2 and 2.5 log_10_ (significantly lower than in the other groups) for 72 h.

### Pre-challenge feeding with *B*. *thermophilum* RBL67 limited intestinal lesions

**Fig 5:**
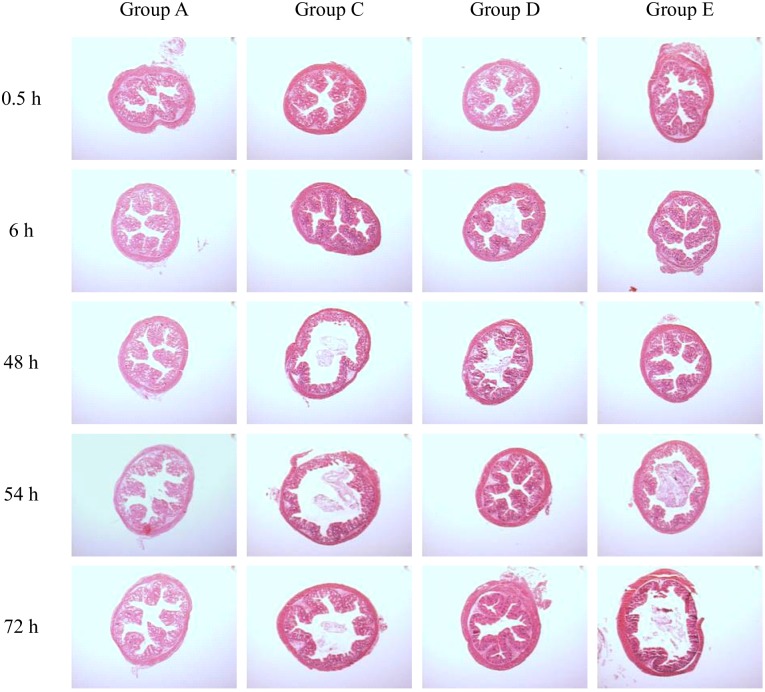
Hematoxylin and eosin stains of cross-sections of colons from suckling CD-1 mice following challenge with simian rotavirus strain SA-11. Group A: not fed strain RBL67, Group C: not fed strain RBL67, challenged with rotavirus SA-11, Group D: fed strain RBL67 before challenge, Group E: fed strain RBL67 after challenge. All images were obtained using the same camera setting and are shown at a magnification of 40X.

Inflammatory histopathology as revealed by hematoxylin and eosin stains of sections of colon from non-infected CD-1 suckling mice (groups A and B) and those challenged with simian rotavirus strain SA-11 with or without *B*. *thermophilum* RBL67 treatment (groups C, D and E) are shown in Fig 5. Colons of group B mice appeared similar to those of control mice (group A). Histological damage was observed in infected mice (group C) beginning 48 h post challenge as was accumulation of fluid in the intestine and dilated colon at necropsy, as described previously [49]. Post-challenge feeding with *B*. *thermophilum* RBL67 (group E) postponed the onset of visible lesions to 54 h. Group D mice showed less epithelial destruction at 6 h and 48 h, and restoration of normal colonic epithelial architecture by 54 h.

### Ingested *Bifidobacterium* strain RBL67 stimulated the humoral immune response in suckling mice

**Fig 6:**
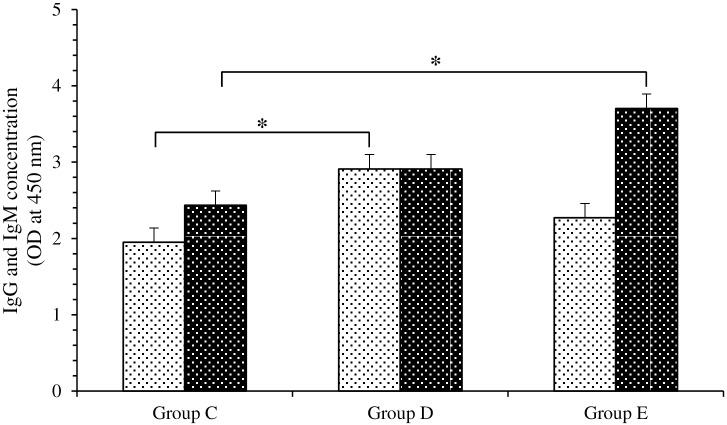
Immunoglobulin G and M production measured (by ELISA) in serum of mice 7 days (light shade) and 14 days (dark shade) after challenge with simian rotavirus strain SA-11. Group C: not fed strain RBL67, challenged with rotavirus SA-11, Group D: fed strain RBL67 before challenge, Group E: fed strain RBL67 after challenge. Error bars indicate the standard error of the mean. An asterisk indicates significantly different from group C (Hsu-Dunnett test, *P* < 0.05, n = 3).

The influence of *B*. *thermophilum* RBL67 on the immune response in suckling CD-1 mice was evaluated by measuring rotavirus-specific IgA in intestinal contents and IgG and IgM in serum (Fig 6). These immunoglobulins were undetectable in mice not challenged with the virus (groups A and B). Four days after challenge with rotavirus (group C), IgA reached 1 OD unit and remained stable until day 7. Ingestion of *B*. *thermophilum* RBL67 (group D) did not change this level significantly. Among group C mice, IgG and IgM reached 2.0 OD on day 7 and rose to 2.4 OD on day 14, and were even higher (2.9 OD) among group D mice (fed *B*. *thermophilum* RBL67 for 1 week prior to challenge) from days 7 through 14. Post-challenge feeding (group E) led to the highest level measured for IgG and IgM (3.7 OD), but not until day 14.

## Discussion

Despite the effectiveness of vaccines against rotavirus, interest in alternative therapies such as probiotics remains keen, due particularly to the high cost of the vaccine and the lack of rotavirus vaccination programs in many developing countries [34, 50, 51]. In the present study, the ability of the probiotic candidate *B*. *thermophilum* RBL67 to inhibit rotavirus infection was assessed *in vitro* using intestinal cell lines and then *in vivo* using suckling mice.

Prior to *in vivo* study, the probiotic candidate should be screened for safety, resistance to gastric acidity and bile acid, adherence to human epithelial cells, and ability to reduce pathogen adhesion [19]. The long history of consumption of fermented milk and the growing body of knowledge on bifidobacteria taxonomy and physiology support the safety of the proposed use of bacteria such as *B*. *thermophilum* RBL67 [52]. This strain has been shown resistant to acidity [32] and should be resistant to bile since it possesses bile salt hydrolase [40]. We evaluated its adhesiveness (Fig 1) to two intestinal cell lines that present different functional characteristics, namely Caco-2 and HT-29 [53]. Its adhesion index on Caco-2 cells is similar to that of *B*. *animalis* subsp. *lactis* Bb12 [54] and *B*. *pseudolongum* ATCC 25526, which are considered highly adhesive [48]. The adhesion index of *B*. *thermophilum* RBL67 was also higher than that of *Lb*. *rhamnosus* GG (the most studied probiotic), estimated at 145 [55]. This ability might be associated in part with the presence of enolase and transaldolase genes, revealed by sequencing of the *B*. *thermophilum* RBL67 genome [40], which are involved in interaction processes with the host [56, 57].

An *in vitro* assay carried out to evaluate to what extent *B*. *thermophilum* RBL67 interfered with rotavirus attachment to intestinal cell lines revealed that this strain excludes rotavirus competitively. Among the three strategies tested, exclusion (contact with the cells for 30 min prior to challenge with the virus) and competition led to the greatest reductions in rotavirus attachment (Fig 2). The exclusion scenario simulates the presence of the probiotic in the intestinal lumen due to ingestion of bacteria as a supplement before infection. Rotavirus attachment was reduced by 2.0 ± 0.1 log_10_ and 1.5 ± 0.1 log_10_ in exclusion and competition assays respectively. To our knowledge, no such reduction has been reported previously in the literature. Reductions in the range of 9.0–49.8% (i.e. less than 1 log_10_) have been observed previously for human rotavirus Wa attachment to MA-104 and HT-29 cells in the presence of 6 *Bifidobacterium* strains, whether in exclusion or competition assays [58], and in the range of 3.3–38% for attachment to Vero cells in the presence of 5 *Bifidobacterium* or 6 *Lactobacillus* strains [59]. In the present study, the third assay (displacement) simulating post-infection feeding led to negligible reduction of rotavirus attachment (0.2 ± 0.1 log_10_), possibly explaining the poor health observed in group E mice.

Based on these results, an *in vivo* study was performed using suckling mice to evaluate the host-protective properties of *B*. *thermophilum* RBL67 and to determine whether or not daily intake could mitigate the risk of rotavirus infection. CD-1 suckling mice were chosen because of their susceptibility to rotavirus (induction of diarrhea and spread of the virus throughout the tissues in a reproducible manner) and they are well characterized, low cost, and easy to handle in large numbers [60, 61]. Ingestion of *B*. *thermophilum* RBL67 increased significantly the concentration of viable *Bifidobacterium* in the intestine, confirming that this probiotic candidate of fecal origin survived the various digestive stresses. The strain persisted in the intestine only for 2 days after daily consumption ceased (Fig 3). Although *B*. *thermophilum* RBL67 has been shown to grow in a simulated human juvenile intestinal environment [38], it did not likely persist in the intestinal microbiota of the suckling mice, since an indigenous population of *Bifidobacterium* was already present. Tannock, Munro [62] demonstrated that a stable population of lactobacilli prevented long-term colonization by *Lb*. *rhamnosus* DR20 ingested as a probiotic. In addition, *B*. *thermophilum* RBL67 did not induce any obvious adverse or toxic effect in suckling CD-1 mice.

Rotavirus induced diarrhea in unprotected mice (group C) over a period of 3 days, starting at 24–30 h post challenge. The onset of this symptom coincided with the increase of viral titer in the intestinal contents (Fig 4) and the appearance of lesions in the intestinal epithelium (Fig 5). As expected [63, 64], the infection triggered the production of rotavirus-specific intestinal IgA and humoral IgG and IgM. Daily ingestion of *B*. *thermophilum* RBL67 prior to infection (group D) had a moderate but clear protective effect, decreasing diarrhea and virus replication for at least 3 days. Such delays in the onset of diarrhea have been reported in association with other probiotic candidates such as *B*. *longum* [59] and *Lb*. *rhamnosus* GG [65]. In contrast, the published data concerning the control of virus shedding are discordant since this is likely strain-specific. *B*. *longum* [59], *Lb*. *rhamnosus* GG [65], *Lb*. *acidophilus* NCFM and *Lb*. *reuteri* ATCC 23272 [66] were reported to have no effect, unlike a combination of *Lb*. *rhamnosus* GG and *B*. *animalis* subsp. *lactis* Bb12, which controlled virus shedding until 4 days post challenge [67]. A significant initial reduction in rotavirus shedding has been noted following pre-challenge feeding with *B*. *longum* subsp. *infantis* CECT 7210, but this was not maintained beyond day 2 post challenge [58]. Daily ingestion of *B*. *thermophilum* RBL67 prior to challenge reduced damage to the intestinal epithelium, which recovered its normal architecture by 54 h post challenge. Some preservation of vacuolated enterocytes has been associated with *Lb*. *rhamnosus* GG, although normal architecture was still not restored 6 days post challenge [65]. Post-challenge feeding with *B*. *thermophilum* RBL67 (group E) provided less conclusive results than did pre-challenge feeding (group D), in which the increase in intestinal rotavirus titer and damage to the epithelium were limited to the 30–54 h period before returning to levels observed in control mice (group B). Nevertheless, these effects are similar to those observed with pre-challenge ingestion of *B*. *longum* subsp. *infantis* CECT 7210 [58].

Several mechanisms have been proposed to explain the efficacy of probiotics in the prevention and treatment of diarrheal diseases. The possible mechanisms include among others production of acidity, short-chain fatty acids and antimicrobial substances, normalization of perturbed microbiota, increased turnover of enterocytes, competitive exclusion of pathogens, improved barrier function, and stimulation of immune responses to pathogens [20, 68]. The moderate protective effect associated with *B*. *thermophilum* RBL67 intake could be due partly to competitive exclusion of rotavirus, tight junction strengthening [39] and stimulation of the immune response. Indeed, recovery from infection is correlated primarily with rotavirus-specific antibody production [69]. We found no significant stimulation of IgA in response to ingestion of *B*. *thermophilum* RBL67. Regarding this immunoglobulin, conflicting results have been reported: some researchers have observed stimulation [70–73] while others did not [58]. However, ingestion of *B*. *thermophilum* RBL67 was associated with an obvious specific humoral response reflected in IgG and IgM (Fig 6). This was accelerated when *B*. *thermophilum* RBL67 was already present in the intestinal lumen at the time of challenge compared to when it was ingested post challenge. Others have shown that probiotic candidates *Lb*. *acidophilus* NCFM and *Lb*. *reuteri* ATCC 23272 increase total small intestinal IgM and IgG levels in rotavirus-infected gnotobiotic piglets [66]. These results suggest that the immunomodulatory effect of a probiotic is strain-specific [74]. Although IgA is thought to provide the most effective protection against rotavirus in the intestine, evidence suggests that serum IgG or IgM in sufficient quantities can reach the intestinal epithelium and provide additional protection [69, 75–77]. This study does not exclude other modalities of host immune response, in particular the effect on cytokine-secreting cells [78], which should be investigated in a future study.

In summary, the probiotic candidate *Bifidobacterium thermophilum* strain RBL67 reduced the duration of diarrhea, limited epithelial lesions, controlled rotavirus replication in the intestine, stimulated the humoral specific IgG and IgM response, and shortened the time of recovery from symptoms in CD-1 suckling mice. Furthermore, *B*. *thermophilum* RBL67 displayed high adhesiveness and competitiveness and hence interference with rotavirus attachment to intestinal cells *in vitro*. These functions might contribute to the mechanisms underlying the moderate protective effect of *B*. *thermophilum* RBL67 against rotavirus in this model of infection. In addition, *B*. *thermophilum* RBL67 meets several criteria for probiotic bacteria: presumed safety, resistance to gastric acidity and bile acids, adherence to human epithelial cells, ability to reduce virus attachment and to reduce the symptoms of infection in suckling mice, and its genome now fully sequenced for scientific scrutiny. Further studies are required in order to identify the mechanisms underlying the apparent beneficial effects of *B*. *thermophilum* RBL67 and to confirm our results in a more human-like model (such as piglets) before proceeding with human trials. If these beneficial effects are also observed in human, a prophylaxis use of *B*. *thermophilum* RBL67 coupled with supplementary or complementary treatment during the winter seasonal peaks of gastroenteritis could be envisaged in children.

## Supporting Information

S1 Fig Attachment of human rotavirus strain Wa to intestinal cells: (A) Caco-2, (B) HT-29. Monolayers were contacted with rotavirus suspended at a titer of 6.9 log_10_ ffu/mL and attachment was measured directly by immunofluorescence assay. Error bars indicate the standard error of the mean. Different letters indicate significant difference between assay times (Tukey’s HSD test, P < 0.05, n = 3).
(DOCX)

S2 Fig Average body weight of CD-1 suckling mice during 7 days after challenge with rotavirus SA-11: not fed strain RBL67 (group A, ◆), fed strain RBL67 (group B, ▴), not fed strain RBL67, challenged with rotavirus SA-11 (group C, ✕), fed strain RBL67 before challenge (group D, ■), fed strain RBL67 after challenge (group E, ●) (See Table 1 for details). Error bars indicate the standard error of the mean. An asterisk indicates statistical significance compared to **◆** (Tukey’s HSD test, P < 0.05, n = 189).
(DOCX)

S1 File Data. (PDF)
