# Supplementary material for: Study of the Ability of Bifidobacteria of Human Origin to Prevent and Treat Rotavirus Infection Using Colonic Cell and Mouse Models
Source: PLoS One. 2016 Oct 11;11(10):e0164512. doi: 10.1371/journal.pone.0164512 (PMC5058500; doi:10.1371/journal.pone.0164512)
Supplement: S1 File — (PDF) [file pone.0164512.s003.pdf]

Fig_1

Adhesion index to Caco-2 cells

Level	Rep 1	Rep 2	Rep 3	Rep 4	Mean	Standard error
RBL67	625	625	500	750	625	85
RBL69	938	288	500	ND	575	98
RBL70	813	750	875	ND	813	98
ATCC 15707	66	55	43	ND	54	98
ATCC 25526	469	375	563	ND	469	170

Adhesion index to HT-29 cells

Level	Rep 1	Rep 2	Rep 3	Rep 4	Mean	Standard error
RBL67	3250	1188	1438	1958	1958	319
RBL69	688	875	779	ND	781	390
RBL70	1188	1225	1204	ND	1206	390
ATTC 15707	175	113	163	ND	150	319
ATCC 25526	438	813	995	ND	748	276

Fig_2

Rotavirus attachment to Caco-2 cells (log10 ffu/mL)

Level	Rep 1	Rep 2	Rep 3	Mean	Standard error
Positive control	5,81	5,81	ND	5,81	0,05
Exclusion	3,94	3,69	3,84	3,83	0,05
Competition	4,20	4,40	4,41	4,33	0,05
Displacement	5,57	5,58	5,59	5,58	0,05

Rotavirus attachment to HT-29 cells (log10 ffu/mL)

Level	Rep 1	Rep 2	Rep 3	Mean	Standard error
Positive control	5,76	5,76	ND	5,76	0,05
Exclusion	3,94	3,67	3,84	3,82	0,05
Competition	4,41	4,40	4,20	4,33	0,05
Displacement	5,53	5,48	5,51	5,51	0,05

Fig_3

Concentrations of viable *Bifidobacterium* in intestinal content (log10 cfu/g)

Group	Time	Rep 1	Rep 2	Rep 3	Mean	Standard error
A	0,5	3,59	3,67	ND	3,63	0,11
A	6	3,91	3,85	ND	3,88	1,00
A	24	3,89	3,97	ND	3,93	0,54
A	30	4,06	4,10	ND	4,08	0,48
A	48	3,96	4,00	ND	3,98	0,51
A	54	3,93	3,89	ND	3,91	0,50
A	72	3,95	3,85	ND	3,90	0,15
B	0,5	7,75	7,39	ND	7,57	0,08
B	6	6,35	5,90	ND	6,12	0,71
B	24	5,24	3,96	ND	4,60	0,38
B	30	5,78	4,55	ND	5,16	0,34
B	48	5,57	4,18	ND	4,87	0,36
B	54	4,26	4,88	ND	4,57	0,35
B	72	4,27	4,26	ND	4,27	0,15
C	0,5	4,17	4,24	ND	4,21	0,08
C	6	4,18	3,81	ND	3,99	0,71
C	24	4,28	3,76	ND	4,02	0,38
C	30	4,34	4,31	ND	4,33	0,34
C	48	4,63	4,02	ND	4,33	0,36
C	54	4,20	4,00	ND	4,10	0,35
C	72	4,27	4,04	ND	4,16	0,10
D	0,5	7,70	7,75	7,62	7,69	0,06
D	6	5,58	7,13	6,68	6,46	0,58
D	24	6,08	6,32	6,82	6,40	0,31
D	30	6,01	6,41	5,70	6,04	0,28
D	48	4,74	4,90	5,18	4,94	0,30
D	54	5,17	4,11	5,38	4,89	0,29
D	72	4,51	4,31	4,46	4,42	0,08
E	0,5	4,39	4,39	ND	4,39	0,06
E	6	4,02	6,46	6,46	5,64	0,58
E	24	6,28	7,12	6,36	6,59	0,31
E	30	7,18	6,99	6,28	6,82	0,28
E	48	7,08	6,71	6,16	6,65	0,30
E	54	6,43	5,89	5,48	5,93	0,29
E	72	6,32	6,27	5,87	6,15	0,08

Fig_4

Concentrations of rotavirus SA-11 in intestinal content (log10 pfu/g)

Group	Time	Rep 1	Rep 2	Mean	Standard error
C	0,5	3,00	3,00	3,00	0,00
C	3	2,70	2,70	2,70	0,00
C	6	3,03	2,70	2,86	0,13
C	24	4,06	3,78	3,92	0,08
C	30	5,49	4,90	5,20	0,20
C	48	4,35	4,00	4,17	0,18
C	72	4,40	4,08	4,24	0,20
D	0,5	2,83	2,83	2,83	0,00
D	3	2,48	2,48	2,48	0,00
D	6	2,74	2,44	2,59	0,13
D	24	2,79	2,72	2,76	0,08
D	30	2,92	2,53	2,72	0,20
D	48	3,46	2,94	3,20	0,18
D	72	2,13	2,76	2,45	0,20
E	0,5	2,89	2,89	2,89	0,00
E	3	2,30	2,30	2,30	0,00
E	6	2,97	2,97	2,97	0,13
E	24	3,23	3,23	3,23	0,08
E	30	3,48	3,48	3,48	0,20
E	48	4,63	4,63	4,63	0,18
E	72	4,39	4,39	4,39	0,20

Fig_6

IgA levels

Group	Time	Rep 1	Rep 2	Rep 3	Mean	Standard error
C	4	0,70	1,00	1,00	0,90	0,17
C	7	1,00	1,15	0,98	1,04	0,09
D	4	1,00	1,30	1,00	1,10	0,17
D	7	1,00	1,30	1,00	1,10	0,17
E	4	0,70	1,00	1,60	1,10	0,46
E	7	0,70	1,30	1,30	1,10	0,35

IgG and IgM levels

Group	Time	Rep 1	Rep 2	Rep 3	Mean	Standard error
C	7	1,85	1,95	2,05	1,95	0,19
C	14	2,56	2,42	2,32	2,43	0,19
D	7	3,39	2,43	2,91	2,91	0,19
D	14	3,39	2,43	2,91	2,91	0,19
E	7	2,43	2,43	1,95	2,27	0,19
E	14	3,86	3,39	3,86	3,70	0,19

S1_Fig

Rotavirus attachment to Caco-2 cells (log10 ffu/mL)

Time	Rep 1	Rep 2	Rep 3	Mean	Standard error
1,5	6,34	6,24	5,96	6,18	0,08
3	6,16	6,24	6,16	6,19	0,08
6	5,34	5,18	5,24	5,25	0,08
24	4,34	4,30	4,10	4,25	0,08
48	3,60	3,80	4,01	3,80	0,08

Rotavirus attachment to HT-29 cells (log10 ffu/mL)

Time	Rep 1	Rep 2	Rep 3	Mean	Standard error
1,5	6,34	6,01	6,01	6,12	0,06
3	6,10	6,18	6,24	6,17	0,06
6	5,34	5,24	5,30	5,30	0,06
24	3,80	3,60	3,80	3,73	0,06
48	3,34	3,30	3,24	3,30	0,06

S2_Fig

Changes in weight of CD-1 suckling mice (g)

		Rep 1													Rep 2				
Group	Time	Mouse 1	Mouse 2	Mouse 3	Mouse 4	Mouse 5	Mouse 6	Mouse 7	Mouse 8	Mouse 9	Mouse 10	Mouse 11	Mouse 12	Mouse 13	Mouse 1	Mouse 2	Mouse 3	Mouse 4	Mouse 5
A	0	6,18	6,76	6,50	6,24	6,92	6,94	7,11	6,73	6,05	6,08	6,76	6,42	6,54	6,10	6,07	6,14	6,36	5,88
A	1	7,06	6,92	7,01	6,87	6,61	6,42	6,85	6,91	7,29	6,45	7,03	6,31	6,40	6,46	6,56	6,84	6,65	6,78
A	2	7,73	7,52	7,58	7,80	7,95	7,52	7,66	8,07	7,70	8,03	7,41	7,47	7,65	7,49	7,41	7,69	6,88	7,61
A	3	8,60	8,35	8,59	8,50	8,85	8,92	8,49	8,92	8,30	8,94	8,20	8,92	8,10	7,61	8,44	8,16	8,06	8,53
A	4	8,66	9,20	8,80	9,03	8,00	8,44	8,21	8,92	9,10	9,10	8,60	8,83	8,80	8,66	7,86	8,49	8,44	8,44
A	5	10,01	9,39	10,10	9,65	9,02	9,53	10,07	9,31	9,22	9,62	9,47	9,90	9,36	8,80	9,11	8,76	9,17	8,24
A	6	9,40	9,47	9,71	9,38	9,27	9,77	10,30	9,90	9,85	9,67	9,43	10,16	9,67	9,69	9,59	8,73	9,21	9,14
A	7	9,97	9,70	10,30	9,70	10,21	9,95	10,15	9,77	9,97	10,25	9,50	9,80	10,03	9,30	9,68	9,55	9,84	9,47
B	0	7,97	8,09	7,81	7,68	7,70	7,64	–	–	–	–	–	–	–	6,22	6,54	6,60	6,87	6,14
B	1	8,60	8,67	8,56	8,80	–	–	–	–	–	–	–	–	–	7,41	7,49	7,02	7,12	6,70
B	2	9,52	–	–	–	–	–	–	–	–	–	–	–	–	8,71	8,70	8,36	8,33	8,02
B	3	9,60	–	–	–	–	–	–	–	–	–	–	–	–	9,55	9,90	9,13	9,22	9,33
B	4	–	–	–	–	–	–	–	–	–	–	–	–	–	10,34	9,84	9,89	9,78	–
B	5	–	–	–	–	–	–	–	–	–	–	–	–	–	10,88	10,26	10,52	10,61	–
B	6	–	–	–	–	–	–	–	–	–	–	–	–	–	10,54	10,84	10,35	10,59	–
B	7	–	–	–	–	–	–	–	–	–	–	–	–	–	10,24	10,56	10,66	10,31	–
C	0	5,72	5,65	5,68	5,80	5,60	5,54	5,74	5,50	5,91	–	–	–	–	5,98	5,88	6,29	5,78	5,48
C	1	6,25	6,19	6,06	6,42	6,10	6,15	6,27	6,33	–	–	–	–	–	6,48	5,82	6,02	5,79	6,21
C	2	7,12	7,12	6,75	6,78	7,36	7,01	–	–	–	–	–	–	–	6,58	6,52	7,29	6,61	6,81
C	3	7,51	8,19	7,86	7,87	–	–	–	–	–	–	–	–	–	7,92	7,64	8,40	7,28	7,29
C	4	8,27	8,42	8,74	–	–	–	–	–	–	–	–	–	–	9,30	8,05	9,08	8,19	8,55
C	5	8,31	8,71	–	–	–	–	–	–	–	–	–	–	–	9,69	10,11	8,73	9,27	9,77
C	6	8,08	8,26	–	–	–	–	–	–	–	–	–	–	–	10,54	10,37	9,50	9,98	9,08
C	7	7,91	7,82	–	–	–	–	–	–	–	–	–	–	–	11,56	10,29	10,87	11,42	10,27
D	0	5,59	5,11	5,43	5,58	5,42	5,14	5,43	5,52	5,48	5,84	5,16	–	–	6,59	6,27	6,15	6,47	5,86
D	1	6,43	5,94	5,66	5,80	5,66	5,69	5,94	5,84	5,59	5,97	–	–	–	7,26	6,73	7,21	7,22	6,75
D	2	6,48	6,67	6,58	6,30	6,48	6,33	6,27	6,91	–	–	–	–	–	8,08	7,80	8,00	7,49	7,61
D	3	7,19	7,43	7,67	7,58	7,55	7,01	–	–	–	–	–	–	–	9,13	9,27	8,98	9,26	8,43
D	4	8,60	8,69	8,04	8,13	8,34	–	–	–	–	–	–	–	–	9,98	10,22	10,17	10,11	9,52
D	5	9,10	9,40	8,44	8,99	8,64	–	–	–	–	–	–	–	–	10,74	10,92	11,17	10,67	10,24
D	6	8,64	9,10	9,52	9,56	9,36	–	–	–	–	–	–	–	–	11,73	11,50	10,74	11,20	11,08
D	7	9,36	9,99	10,02	9,31	–	–	–	–	–	–	–	–	–	11,43	10,81	12,03	11,42	11,50
E	0	6,29	6,45	6,92	6,10	5,90	5,93	6,65	6,31	6,40	6,20	–	–	–	4,97	4,50	4,07	4,01	4,13
E	1	6,59	6,98	7,19	7,01	6,32	6,61	6,60	7,00	6,82	–	–	–	–	4,58	4,42	4,13	3,94	4,74
E	2	8,13	7,37	7,76	7,87	7,56	7,40	7,76	–	–	–	–	–	–	6,41	4,84	4,59	4,93	5,05
E	3	8,77	8,51	8,69	9,11	8,86	–	–	–	–	–	–	–	–	5,90	5,61	5,69	6,81	5,37
E	4	9,88	9,27	10,02	9,75	–	–	–	–	–	–	–	–	–	5,68	7,00	5,82	5,08	5,88
E	5	9,75	10,88	10,63	10,46	–	–	–	–	–	–	–	–	–	6,12	6,15	6,15	6,09	7,29
E	6	11,05	10,31	10,98	10,93	–	–	–	–	–	–	–	–	–	6,40	6,55	6,03	6,46	6,51
E	7	11,48	11,72	11,48	–	–	–	–	–	–	–	–	–	–	7,00	6,77	8,01	7,10	6,25

(S2_Fig continued)

		Rep 2 (continued)													Rep 3				
Group	Time	Mouse 6	Mouse 7	Mouse 8	Mouse 9	Mouse 10	Mouse 11	Mouse 12	Mouse 13	Mouse 14	Mouse 15	Mouse 16	Mouse 17	Mouse 18	Mouse 1	Mouse 2	Mouse 3	Mouse 4	Mouse 5
A	0	6,09	6,54	6,03	–	–	–	–	–	–	–	–	–	–	6,44	7,60	7,14	6,80	6,53
A	1	6,51	6,50	6,21	–	–	–	–	–	–	–	–	–	–	7,29	7,46	8,00	7,75	7,12
A	2	7,37	7,30	–	–	–	–	–	–	–	–	–	–	–	7,82	8,11	8,25	8,36	9,00
A	3	8,07	8,27	–	–	–	–	–	–	–	–	–	–	–	8,81	9,25	9,91	8,71	–
A	4	8,28	8,94	–	–	–	–	–	–	–	–	–	–	–	9,90	10,47	9,48	–	–
A	5	8,74	8,61	–	–	–	–	–	–	–	–	–	–	–	10,78	9,65	9,99	–	–
A	6	9,63	9,11	–	–	–	–	–	–	–	–	–	–	–	9,67	10,32	10,80	–	–
A	7	8,72	9,93	–	–	–	–	–	–	–	–	–	–	–	10,94	11,51	9,84	–	–
B	0	6,28	6,96	6,70	6,86	7,18	6,69	–	–	–	–	–	–	–	–	–	–	–	–
B	1	7,54	7,61	7,47	7,73	–	–	–	–	–	–	–	–	–	–	–	–	–	–
B	2	8,05	7,95	–	–	–	–	–	–	–	–	–	–	–	–	–	–	–	–
B	3	–	–	–	–	–	–	–	–	–	–	–	–	–	–	–	–	–	–
B	4	–	–	–	–	–	–	–	–	–	–	–	–	–	–	–	–	–	–
B	5	–	–	–	–	–	–	–	–	–	–	–	–	–	–	–	–	–	–
B	6	–	–	–	–	–	–	–	–	–	–	–	–	–	–	–	–	–	–
B	7	–	–	–	–	–	–	–	–	–	–	–	–	–	–	–	–	–	–
C	0	4,34	5,38	5,43	6,29	6,13	6,11	5,62	–	–	–	–	–	–	5,93	5,63	5,97	5,95	5,57
C	1	6,73	6,86	6,53	4,94	6,06	6,64	–	–	–	–	–	–	–	6,11	6,67	6,61	6,40	6,48
C	2	7,47	6,85	5,61	7,47	–	–	–	–	–	–	–	–	–	7,18	7,26	6,44	7,11	6,46
C	3	7,22	8,31	–	–	–	–	–	–	–	–	–	–	–	7,26	7,10	8,19	7,35	8,26
C	4	8,94	–	–	–	–	–	–	–	–	–	–	–	–	9,11	9,09	9,01	9,02	8,88
C	5	8,55	–	–	–	–	–	–	–	–	–	–	–	–	9,17	9,75	8,88	9,96	9,88
C	6	10,25	–	–	–	–	–	–	–	–	–	–	–	–	10,43	10,66	10,60	10,65	9,86
C	7	–	–	–	–	–	–	–	–	–	–	–	–	–	11,24	10,66	11,13	11,09	10,21
D	0	6,44	6,46	6,66	5,53	6,66	5,98	6,06	–	–	–	–	–	–	5,09	4,80	5,20	4,90	4,34
D	1	6,77	7,14	7,26	7,31	6,45	–	–	–	–	–	–	–	–	5,30	4,79	5,61	5,36	5,84
D	2	8,18	8,16	7,26	–	–	–	–	–	–	–	–	–	–	6,07	6,52	5,42	6,12	5,00
D	3	9,22	–	–	–	–	–	–	–	–	–	–	–	–	5,91	6,78	6,88	6,45	6,89
D	4	–	–	–	–	–	–	–	–	–	–	–	–	–	7,55	6,71	7,90	7,30	7,20
D	5	–	–	–	–	–	–	–	–	–	–	–	–	–	8,52	8,10	7,18	8,02	8,19
D	6	–	–	–	–	–	–	–	–	–	–	–	–	–	8,93	8,36	7,74	8,65	7,34
D	7	–	–	–	–	–	–	–	–	–	–	–	–	–	8,34	9,19	8,93	7,85	9,30
E	0	5,59	4,66	4,13	4,24	4,29	3,82	3,8	3,97	4,05	4,08	4,15	3,93	4,03	6,11	5,97	6,68	5,40	6,55
E	1	4,00	4,18	4,70	5,54	4,17	4,84	5,67	4,34	4,54	4,51	4,30	–	–	7,15	7,22	6,64	6,79	6,50
E	2	5,19	4,48	4,40	5,04	4,91	4,69	5,83	4,88	5,22	–	–	–	–	6,78	8,02	8,09	8,01	7,25
E	3	5,00	4,76	5,38	5,19	5,44	5,51	–	–	–	–	–	–	–	8,98	7,73	8,33	9,09	8,41
E	4	5,68	6,00	5,61	5,39	5,65	5,91	–	–	–	–	–	–	–	10,01	9,44	10,14	8,94	9,34
E	5	5,40	6,22	5,97	6,02	5,61	6,08	–	–	–	–	–	–	–	10,13	10,85	10,45	9,38	10,10
E	6	6,05	6,59	6,55	6,72	6,49	7,69	–	–	–	–	–	–	–	10,40	10,90	11,19	10,84	10,07
E	7	7,08	6,99	6,66	6,98	7,25	7,02	–	–	–	–	–	–	–	10,67	10,88	11,98	11,74	11,32

(S2_Fig continued)

		Rep 3 (continued)											
Group	Time	Mouse 6	Mouse 7	Mouse 8	Mouse 9	Mouse 10	Mouse 11	Mouse 12	Mouse 13	Mouse 14	Mouse 15	Mean	Standard error
A	0	6,90	7,08	6,92	6,70	7,06	–	–	–	–	–	6,43	0,15
A	1	7,55	7,50	7,43	–	–	–	–	–	–	–	6,75	0,17
A	2	7,82	–	–	–	–	–	–	–	–	–	7,62	0,19
A	3	–	–	–	–	–	–	–	–	–	–	8,47	0,25
A	4	–	–	–	–	–	–	–	–	–	–	8,64	0,31
A	5	–	–	–	–	–	–	–	–	–	–	9,37	0,34
A	6	–	–	–	–	–	–	–	–	–	–	9,57	0,34
A	7	–	–	–	–	–	–	–	–	–	–	9,84	0,37
B	0	–	–	–	–	–	–	–	–	–	–	7,21	0,15
B	1	–	–	–	–	–	–	–	–	–	–	7,82	0,20
B	2	–	–	–	–	–	–	–	–	–	–	8,61	0,28
B	3	–	–	–	–	–	–	–	–	–	–	9,46	0,42
B	4	–	–	–	–	–	–	–	–	–	–	9,96	0,59
B	5	–	–	–	–	–	–	–	–	–	–	10,57	0,67
B	6	–	–	–	–	–	–	–	–	–	–	10,58	0,67
B	7	–	–	–	–	–	–	–	–	–	–	10,44	0,72
C	0	5,39	6,35	5,86	6,07	6,33	6,11	5,98	5,22	5,16	–	5,75	0,15
C	1	5,93	6,56	5,63	6,22	6,50	6,75	5,80	–	–	–	6,16	0,17
C	2	7,22	7,33	6,33	7,25	–	–	–	–	–	–	6,90	0,19
C	3	8,00	8,00	8,41	–	–	–	–	–	–	–	7,77	0,25
C	4	8,19	8,11	–	–	–	–	–	–	–	–	8,58	0,31
C	5	9,74	9,99	–	–	–	–	–	–	–	–	9,23	0,36
C	6	9,86	10,73	–	–	–	–	–	–	–	–	9,40	0,36
C	7	11,02	10,90	–	–	–	–	–	–	–	–	10,02	0,40
D	0	5,41	5,11	4,63	5,61	5,26	5,14	3,94	5,58	5,13	4,53	5,69	0,15
D	1	4,19	4,63	5,54	5,55	5,85	6,10	5,05	5,29	–	–	6,25	0,17
D	2	6,80	6,34	6,45	5,86	5,78	–	–	–	–	–	7,00	0,19
D	3	6,75	7,15	5,81	6,92	–	–	–	–	–	–	8,14	0,25
D	4	6,43	7,66	7,44	–	–	–	–	–	–	–	9,18	0,30
D	5	8,16	7,66	6,90	–	–	–	–	–	–	–	9,83	0,33
D	6	8,03	8,63	8,68	–	–	–	–	–	–	–	10,24	0,33
D	7	8,89	8,11	8,78	–	–	–	–	–	–	–	10,65	0,37
E	0	5,57	5,87	6,44	6,63	6,12	5,80	5,96	5,54	–	–	5,66	0,15
E	1	7,24	5,84	6,13	6,48	6,79	6,60	–	–	–	–	5,92	0,17
E	2	6,82	7,67	7,36	7,39	–	–	–	–	–	–	6,64	0,19
E	3	8,11	–	–	–	–	–	–	–	–	–	7,33	0,25
E	4	8,77	–	–	–	–	–	–	–	–	–	7,60	0,31
E	5	9,40	–	–	–	–	–	–	–	–	–	8,17	0,34
E	6	11,59	–	–	–	–	–	–	–	–	–	8,36	0,34
E	7	11,42	–	–	–	–	–	–	–	–	–	8,73	0,38
